# Safe Routes to Age in Place: A Focus on Alternative Transportation

**DOI:** 10.1093/geroni/igab046.2139

**Published:** 2021-12-17

**Authors:** Holly Dabelko-Schoeny, Noelle Fields, Katie White, Christine Highfill, Qiuchang Cao, Ian Murphy

**Affiliations:** 1 The Ohio State University, The Ohio State University, Ohio, United States; 2 University of Texas at Arlington, Arlington, Texas, United States; 3 Ohio State University, Age-Friendly Columbus and Franklin County, Ohio, United States; 4 University of Texas - Arlington, Arlington, Texas, United States; 5 The Ohio State University, Columbus, Ohio, United States

## Abstract

Informed by social cognitive theory, Age-Friendly Columbus and Franklin County conducted a community-engaged mixed methods study that examined the needs and utilization of alternative transportation by older residents in three pilot neighborhoods in Franklin County, Ohio (n = 32). Participants were provided tablets and used an app (MyAmble) developed at the University of Texas-Arlington to document their traveling experiences. During a 14-day period, 1,190 trips were recorded by older adults and 71.3% of these trips were completed through driving their own personal vehicles. Participants designated 84.5% of trips as important and 72% of the trips improved their mood. Individual (physical and cognitive functioning, cost, time), environmental (lighting, sidewalk conditions, traffic, location of bus stops, weather), and behavioral (no history of bus use, peer to peer information sharing, tracking led to future planning) barriers and facilitators to alternative transportation use such as riding the bus, walking and biking were identified.

